# Hypervalency in amorphous chalcogenides

**DOI:** 10.1038/s41467-022-29054-5

**Published:** 2022-03-18

**Authors:** T. H. Lee, S. R. Elliott

**Affiliations:** 1 Department of Chemistry, University of Cambridge, Lensfield Road, Cambridge, CB2 1EW UK; 2 School of Materials Science and Engineering, Kyungpook National University, Daegu, South Korea; 3 Trinity College, Cambridge, CB2 1TQ UK; 4 Department of Chemistry, University of Oxford, Oxford, OX1 3TF UK

**Keywords:** Atomistic models, Electronic materials, Structure of solids and liquids, Molecular dynamics, Density functional theory

## Abstract

The concept of hypervalency emerged as a notion for chemical bonding in molecules to explain the atomic coordination in hypervalent molecules that violates the electron-octet rule. Despite its significance, however, hypervalency in condensed phases, such as amorphous solids, remains largely unexplored. Using ab initio molecular-dynamics simulations, we report here the underlying principles of hypervalency in amorphous chalcogenide materials, in terms of the behaviour of hypervalent structural units, and its implicit relationship with material properties. The origin of a material-dependent tendency towards hypervalency is made evident with the multi-centre hyperbonding model, from which its relationship to abnormally large Born effective charges is also unambiguously revealed. The hyperbonding model is here extended to include interactions with cation *s*^2^ lone pairs (LPs); such deep-lying LPs can also play a significant role in determining the properties of these chalcogenide materials. The role of hypervalency constitutes an indispensable and important part of chemical interactions in amorphous and crystalline chalcogenide solids.

## Abstract (web summary)

The current study explores by ab-initio molecular dynamics simuations the concept of hypervalency in amorphous chalcogenide materials, from which a unified conceptual framework for understanding chemical bonding, microscopic structures, and structure-property relationships is established.

## Introduction

The atomic structures of inorganic glasses are often described in terms of a small number of structural building blocks and with a continuous random-network framework, popularised by the seminal classical work of Zachariasen^1^. It is the presence of well-defined short-range order in these glasses that makes such a description feasible. However, when heavy elements are involved as the main components, the metallic character in the bonding increases, and the coordination chemistry for each element becomes significantly more complicated^2–4^. This is indeed the case for chalcogenide glasses consisting of heavy chalcogen elements. Interesting material systems of this type include, for instance, binary or ternary amorphous tellurides, represented by phase-change memory materials (PCMs)^5–7^, or even monatomic amorphous selenium^8^ or tellurium^9–11^, whose material properties are often significantly influenced by groups of local atomic coordinations, each with characteristically different chemical-bonding properties. For those amorphous materials, typical experimental structural methods, such as Raman spectroscopy^12,13^, extended X-ray absorption fine structure (EXAFS)^14–17^ or nuclear magnetic resonance (NMR) spectroscopy^18,19^, are usually insufficient to provide a detailed description of individual local bonding geometries around atoms, not to mention the effect of each individual type of local structural environment on material properties.

Ab initio molecular-dynamics (AIMD) simulations, based on density-functional theory (DFT), have proven to be well suited for this purpose^2,3,7–10,20–26^. Accordingly, this method has provided useful information on local atomic coordinations from the atomic positions in simulated models of amorphous solids. A representative example would be recent studies on amorphous PCMs^2,3,7,20–26^. However, it turned out that, for a comprehensive understanding of the local bonding geometries and of their relationship with material properties, a sophisticated analysis tool beyond this conventional approach was required. One such proposed methodology is based on the decomposition of complicated electron-charge distributions into bonding and non-bonding (lone pair) electron pairs^4,27^. A theoretical basis for the interpretation of the resultant distributions of the electron pairs was introduced by Lee and Elliott^28^ with the help of the concept of multi-centre hyperbonding. The key element of this bonding theory, distinguishable from normal two-centre, two-electron (2c/2e) covalent bonds, is the involvement of a lone pair in three-centre, four-electron (3c/4e) hyperbonding interactions, whereby a (near-) linearly-aligned triatomic hyperbond pair forms as a consequence of the stabilisation interaction of an occupied LP with a nearby unoccupied antibonding state, in generating a form of dative or coordinate bond^29^. A diversity of interesting material properties of, e.g. GST, can then be elucidated with the aid of this hyperbonding model^28^, including (i) a tendency towards large coordination numbers of cations; (ii) the propensity for short- and long-bond formation in linear triatomic bonding geometries; (iii) abnormally large Born effective charges, and a large optical contrast between amorphous and crystalline phases; (iv) small thermal conductivity of crystalline GST; and v) fast crystallisation behaviour of the amorphous phase. Prior to this hyperbonding model, Popov^30^ and Dembovsky^31^ had similarly employed 3c/4e interactions to understand some bonding configurations in amorphous selenium and some other amorphous materials^32^, in the form of quasi-molecular defects, and Kolobov et al.^33^ investigated vacancy-mediated 3c/4e bond formation in crystalline GST.

In molecules, the concept of hypervalency has been used to discuss those cases when the valence of an atom is different from its lowest stable chemical valence^34^, or, more generally, when the number of bonding and non-bonding (LP) electron pairs associated with a constituent atom exceeds four, violating the electron-octet rule. We adopted the latter definition for the description of structural units in amorphous solids. For instance, the structural units manifesting hypervalency correspond to the (4,1)- or (5,1)-type units of Ge or Sb atoms in *a*-GST^4^, where the first and second numbers in parentheses denote the coordination number and the number of LPs of the central atom, respectively. Their sum exceeds four in both these cases; hence, one can see that hypervalency characterises chemical-bonding features in *a*-GST. Such structural units in similarly hypervalency-active amorphous solids may be generally referred to as hypervalent units, as an analogue of hypervalent molecules for molecular systems, and their presence indicates the occurrence of hypervalency in that material. For a general application of the idea of hypervalency to diverse amorphous materials, however, one needs to address the following unanswered questions, including (i) how to unambiguously define hypervalent units, used here as a synonym for hypervalency, in amorphous materials; (ii) whether the hypervalent local coordination is observed in a diversity of amorphous (chalcogenide) materials; (iii) what is the general rule, if any, governing the manifestation of hypervalent units in amorphous solids; and more importantly, (iv) how are they related to microscopic or macroscopic material properties?

Here, we attempt to address all these questions by thoroughly investigating all types of structural units in various amorphous material systems, including amorphous monatomic chalcogens, binary tellurides, and ternary chalcogenides with different chalcogen elements. Surprisingly, it is shown that hypervalent units are widely observed, even in many of the material systems where the hypervalent configurations have been largely overlooked. Moreover, all the structural building units are comprehensively characterised in terms of three chemical-bonding constituents, i.e. ordinary 2c/2e covalent bonds, 3c/4e hyperbonds, and lone pairs, which fully describe the charge distributions around atoms and their bonding geometries according to the empirical rules of the valence-shell electron-pair repulsion (VSEPR) theory^35^. The relation with the microscopic material property of Born effective charge will be established in this study, and a revised form of the hyperbonding model, involving additional interactions with deep-lying cationic *s*-like LPs, will also be introduced, thereby providing an insight into the structure-property relationships present in the material systems studied here.

## Results

### Amorphous chalcogens

**Fig. 1:**
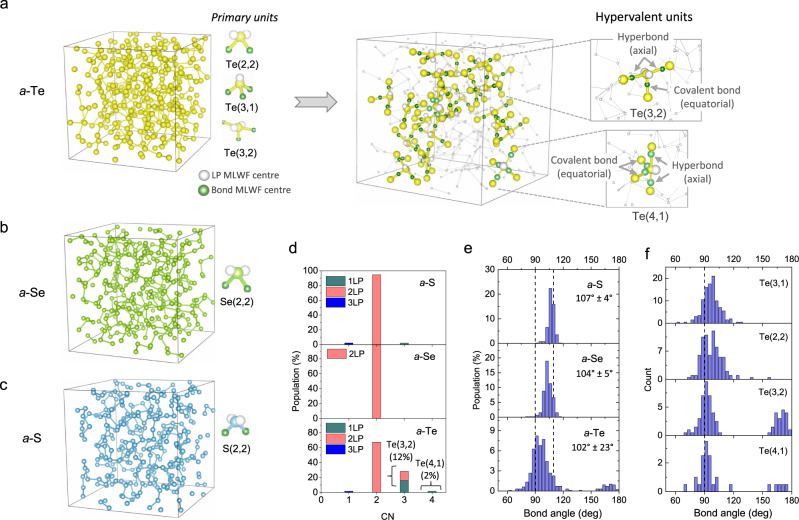
Amorphous chalcogen models and their constituent structural building units. Amorphous models of: **a** *a*-Te; **b** *a*-Se; and **c** *a*-S, and their primary structural units. Each structural unit is specified by two numbers in parentheses, the former denoting the coordination number and the latter the number of LPs associated with the central (chalcogen) atom. For *a*-Te, hyperbonds, covalent bonds and lone pairs (LPs) are specified for each hypervalent unit in **a**. **d** Populations of structural units for each amorphous chalcogen model. The percentages of hypervalent units are noted. **e** Bond-angle distributions (BADs) for structural units found in each model, with the mean bond angle being specified. Bond angles of 90° and 109° are indicated by vertical dotted lines for reference. **f** BADs for various structural units in *a*-Te.

We first consider the simplest chalcogenide material systems, namely monatomic amorphous chalcogens. Figure 1 shows simulated models of amorphous chalcogens (S, Se, Te), together with their constituent structural (building) units. For all the amorphous chalcogen models, the prevailing structural unit is, in general, of the (2,2) type (Fig. 1d), which adopts a bent bonding structure, with two bonds and two LPs being associated with the central chalcogen atom. Most of the chalcogen atoms are involved in forming chains. Structural motifs other than the (2,2) type can be additionally identified in *a*-Te, wherein Te(3,1) (with proportion 16%) and Te(3,2) (12%) units, together with a minor concentration (<2%) of Te(4,1) configurations (Fig. 1a), are also present. The Te(3,2) and Te(4,1) units can be classified as hypervalent units, as their associated number of bonding and non-bonding (LP) electron pairs exceed four, in contrast with the octet-rule-conforming (2,2) or (3,1) types, with four associated bonding and non-bonding electron pairs. Hence, the substantial presence of hypervalent Te(3,2) and Te(4,1) units manifests the pronounced tendency towards hypervalency in *a*-Te. However, this is not the case for the other amorphous chalcogen models of *a*-S and *a*-Se, for which no similar sign of hypervalency (i.e. the presence of hypervalent units) can be identified. All the hypervalent Te(3,2) units in *a*-Te adopt a T-shaped geometry, in which two LPs and one Te ligand occupy equatorial sites, together with two Te ligands bonded along the axial direction, precisely in accord with the prediction of VSEPR theory. Similarly, the hypervalent Te(4,1) unit exhibits a VSEPR-compliant see-saw geometry, where a single LP and two ligands occupy three equatorial sites, along with two axial ligands. For both of these hypervalent units, one (or both) of two end members of the triatomic axial-bond pair often involves a Te(3,1) unit. In fact, such a preferential coordination by Te(3,1) units as a ligand for triatomic axial-bond pairs is commonly observed for other hypervalent units as well (see Supplementary Fig. 1), and is closely in accordance with the scenario of the hyperbonding mechanism, which will be described later in more detail. In any case, it is worth noting at this stage that, in many cases, the population of chalcogen (3,1)-type units provides a measure of the population of hypervalent units in amorphous chalcogen/chalcogenide solids.

More detailed information on the local geometries of those structural motifs can be obtained from their bond-angle distributions (BADs). As shown in Fig. 1e, the average bond angle progressively shifts from the near-tetrahedral bond angle of 107 ± 4° for *a*-S to the smaller angle of 102 ± 23° for *a*-Te. An intermediate mean value of 104 ± 5° is observed for *a*-Se. This gradual decrease in average bond angle may indicate a progressive weakening of *s*-*p* orbital hybridisation towards *p*-type bonding for heavier constituent chalcogen atoms. In fact, for *a*-Te, the situation is more complicated than this simple interpretation suggests, as the BADs are strongly dependent on the type of structural units of interest (Fig. 1f). The hypervalent Te(3,2) and Te(4,1) units show a relatively narrow BAD, centred at ~90°, with a second peak at ~170°: the former peak corresponds to the axial-equatorial or equatorial-equatorial bond angles, while the latter peak represents bond angles between two axial bonds in near-linear triatomic bonding geometries. The angles corresponding to the BAD peaks for the hypervalent units imply that a nearly pure *p*-like character of atomic orbitals (AOs) of a central Te atom is involved in the bonding. In contrast, a larger mean bond angle with a much wider BAD is a distinctive structural signature of the octet-conforming Te(2,2) and Te(3,1) motifs. In particular, the broad BAD for Te(3,1) units arises because the AOs of central Te atoms are involved in two chemically distinct types of bonding, i.e. ordinary 2c/2e covalent and 3c/4e hyperbonding interactions, which will be explored in more detail later in the paper.

### Binary and ternary amorphous chalcogenides

**Fig. 2:**
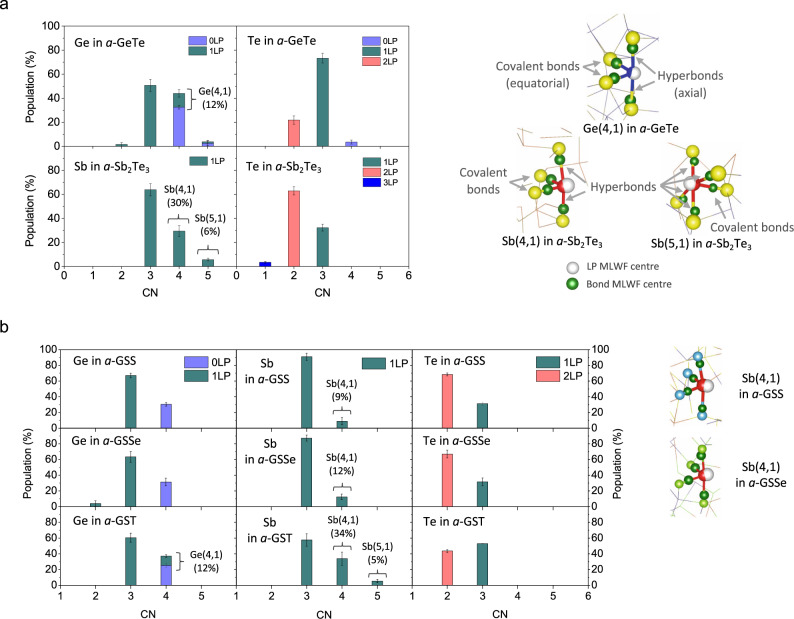
Structural units in binary and ternary amorphous chalcogenides. **a** Populations of structural units in the binary tellurides, *a*-GeTe (upper panels) and *a*-Sb_2_Te_3_ (lower panels). Representative bonding geometries of (4,1)- and (5,1)-type hypervalent units are shown. The positions of MLWF centres for hyperbond pairs, covalent bonds and lone pairs (LPs) are specified for each type of hypervalent unit. **b** Populations of structural units in ternary chalcogenide models of *a*-GSS (top panels), *a*-GSSe (middle panels) and *a*-GST (bottom panels). For *a*-GST, data are taken from Lee and Elliott^4^, with error bars being added. For each model, three independently-generated amorphous model structures were analysed, to obtain better statistics. The standard deviation for those model structures is represented by an error bar.

To gain a more general idea about hypervalency in amorphous solids, binary and ternary amorphous chalcogenides were further investigated. For this purpose, tellurides of germanium (*a*-GeTe) and of antimony (*a*-Sb_2_Te_3_) were chosen as binary alloys, and ternary *a*-Ge_2_Sb_2_S_5_ (*a*-GSS) and *a*-Ge_2_Sb_2_Se_5_ (*a*-GSSe) models were investigated, together with *a*-Ge_2_Sb_2_Te_5_ (*a*-GST) for comparison. All the binary and ternary tellurides studied here belong to a group of PCMs. Figure 2 shows the identified structural units and their relative populations in each model. The data for *a*-GST are taken from Lee and Elliott^4^. For all the models, the largest population of cation units (i.e. Ge or Sb) corresponds to the (3,1)-type unit. For anions (i.e. S, Se, or Te), the (2,2) and (3,1) coordination types constitute the predominant units, as in *a*-Te, although their relative populations depend on the host material. The Te(3,2) units, although abundant in *a*-Te, are seldom found in the binary or ternary chalcogenide models, except in very rare cases when Te atoms happen to be surrounded by Te atoms (see Supplementary Fig. 2); hence, the T-shaped (3,2) geometry represents a characteristic type of unit for Te atoms having mostly Te ligands. There is also a clear dependence on atomic species, such that more hypervalent units are found to be centred on Sb atoms than on Ge, as evidenced by much higher percentages of hypervalent Sb(4,1) than Ge(4,1) units, in general (Fig. 2). The trends observed in ternary chalcogenides with different chalcogen species accord with those observed among the amorphous chalcogen models; that is, the overall tendency towards hypervalency is strongest for the *a*-GST telluride than for the *a*-GSS sulfide or *a*-GSSe selenide models, as inferred from the gradually-decreasing populations of hypervalent Sb(4,1) units in the order of *a*-GST, *a*-GSSe, to *a*-GSS. The populations of chalcogen (3,1) units show the same diminishing trend. The molecular geometries of (4,1)-type units for Ge and Sb atoms precisely correspond to the VSEPR-predicted trigonal bipyramidal structure, as in *a*-Te. Five-fold coordinated Sb(5,1) units, with a square pyramidal structure, are observed only in *a*-Sb_2_Te_3_ and *a*-GST. The (4,0)-type tetrahedral units are found only for Ge atoms, and chalcogen atoms themselves rarely adopt hypervalent configurations in binary or ternary amorphous chalcogenides.

### Structural units and their properties in amorphous solids

So far, we have investigated structural units for each individual material system. As indicated in Figs. 1 and 2, the marked similarities in their shapes (such as in the locations of maximally localised Wannier function (MLWF) centres for LPs/bonds, and in their bond angles) are apparently independent of materials and atomic species, which permits us to approach the problem of hypervalency on a collective basis. To do so, we first classify structural units as either octet-conforming (or simply octet) or non-octet-conforming (or non-octet) types. An octet unit is defined as a structural unit that consists of, in total, four associated bonding and non-bonding electron pairs, while satisfying the Lewis octet rule. Non-octet units are correspondingly defined when the number of associated electron pairs is other than four. In particular, as a subgroup of the non-octet units, any local charge distributions that lead to more than four electron pairs are designated as hypervalent units, as defined previously. The emergence, and degree, of hypervalency in amorphous materials may then be manifested by the presence, and population, respectively, of these hypervalent units.

**Fig. 3:**
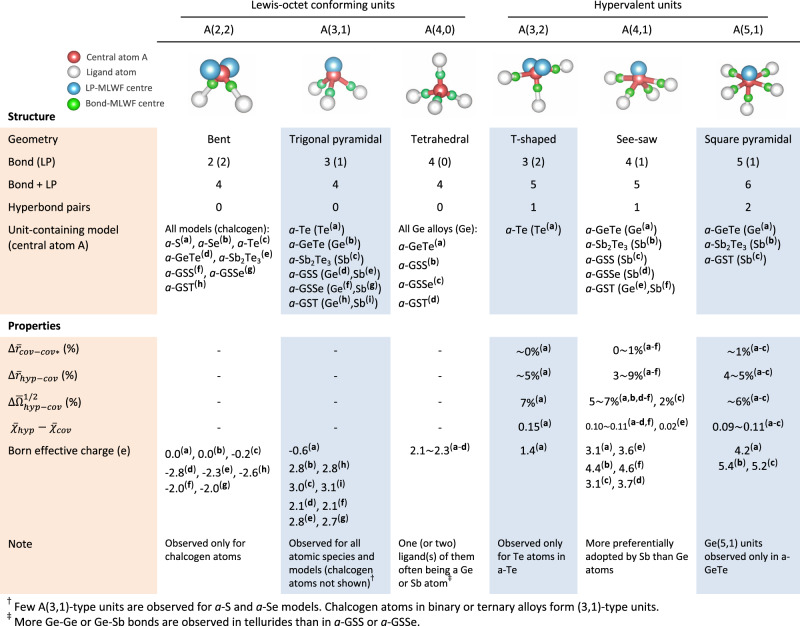
Summary of structural units and their properties in amorphous chalcogens and chalcogenides. Structural units are classified as either a Lewis-octet-conforming unit or a hypervalent unit, depending on whether the sum of their associated numbers of bonds and lone pairs (LPs) is four (octet-conforming units) or more than four (hypervalent units). The positions of MLWF centres, representing either a bond (green) or LP (cyan), are specified for each type of structural unit. Also, for each type of structural unit, a list of models that contain the units in their structure is shown (in the upper panel), together with their corresponding chemical-bonding properties (in the lower panel). The over-strike bar indicates the mean value for the corresponding observables. ${\bar{r}}_{{hyp}}$ (${\bar{r}}_{{cov}}$) denotes the mean bond length of hyperbonds (covalent bonds) for hypervalent units, whilst ${\bar{r}}_{{cov}* }$ indicates the mean bond length of covalent bonds for (2,2)-type units for *a*-Te or for (3,1)-type units for the other models. $\Delta {\bar{r}}_{{cov}-{cov}* }$ stands for the difference between ${\bar{r}}_{{cov}}$ and ${\bar{r}}_{{cov}* }$ in percentage, which is calculated by the equation, $\left({\bar{r}}_{{cov}}/{\bar{r}}_{{cov}* }-1\right)\times 100$. $\Delta {\bar{r}}_{{hyp}-{cov}}$ signifies a difference between ${\bar{r}}_{{hyp}}$ and ${\bar{r}}_{{cov}}$ for the unit, that is, $\left({\bar{r}}_{{hyp}}/{\bar{r}}_{{cov}}-1\right)\times 100$. $\bar{\Omega }$ is the mean quadratic spread of MLWFs^57^ corresponding to hyperbonds (hyp) or covalent bonds (cov) of hypervalent units, while $\Delta {\bar{\Omega }}_{{hyp}-{cov}}^{1/2}$ specifies the difference between ${\bar{\Omega }}_{{hyp}}^{1/2}$ and ${\bar{\Omega }}_{{cov}}^{1/2}$, i.e. $\left({\bar{\Omega }}_{{hyp}}^{1/2}\,/{\bar{\Omega }}_{{cov}}^{1/2}-1\right)\times 100$. $\bar{\chi }$ denotes a polarity index, as defined in Lee and Elliott^4^, quantifying the ionicity of a bond. For homopolar bonds, $\bar{\chi }$ is supposed to be 0.5, and $\bar{\chi }\,$values larger than this value indicate the degree of charge transfer of bonding electrons to a ligand. The Born effective charge (BEC, or Z*) tensor was calculated for the central atom of each structural unit, and one-third of its trace was taken as the BEC value representing each structural unit. The superscripts **a**–**i** specify a correspondence between an atomic species (A) of a material (upper panel) and the bonding properties of its associated structural unit or BEC (lower panel).

Figure 3 summarises the typical structural units found in this study. Three most prevalent types of octet-conforming units can be identified: bent (2,2), trigonal pyramidal (3,1) and tetrahedral (4,0) units. The bent (2,2) and tetrahedral (4,0) units are largely adopted by chalcogen and Ge atoms, respectively. For the latter Ge(4,0) units, in most cases, one or two of the ligands are ‘wrong-bond’ ligands comprising cationic Ge or Sb atoms^24^. Without exception, cations tend to adopt the (3,1)-type unit far more frequently than any other type of unit in amorphous chalcogenides with the compositions considered here. Regarding hypervalent units, it is first noticed that merely a few types of hypervalent units, shown in Fig. 3, are capable of comprehensively describing hypervalency for every model system. The representative hypervalent units are T-shaped (3,2), see-saw (4,1) and square-pyramidal (5,1) units. As shown previously, the T-shaped (3,2) geometry, with a single linear triatomic bonding motif along the axial direction, is mostly adopted by Te atoms in *a*-Te. Apart from the (3,2) unit, the see-saw (4,1) molecular geometry, also containing a single linear triatomic structure, is the most prevalent type of hypervalent unit in amorphous chalcogenides, even including the sulphide^36^ and selenide models (Fig. 2), although the latter non-tellurides apparently prefer octet-conforming local configurations. The (5,1)-type hypervalent units with two perpendicular linear triatomic motifs are more preferentially adopted by Sb atoms than by Ge, yet are much more sparse than Sb(4,1) units. Octahedral coordination with three mutually-perpendicular linear triatomic motifs, which is the maximum coordination allowed for the main-group elements, was seldom found. The data collected over the diversity of material systems and atomic species allow us to conclude that, as a rule of thumb, one can reliably predict any type of structural unit solely based on the number of bonding and non-bonding electron pairs associated with its central atom. The resultant coordination geometry (Fig. 3) then tends to follow the molecular configuration predicted by the VSEPR theory; therefore, a general relationship between the structural units and VSEPR theory emerges in amorphous chalcogenide solids.

Now let us discuss the underlying chemical-bonding interactions associated with each structural unit (Fig. 3). As noted before, any hypervalent unit involves at least one linear triatomic bonding motif, and its presence distinguishes hypervalent units from octet units. An insight into the relevant chemical interactions comes from a recent study^28^ of *a*-GST, in which chemical interactions in *a*-GST were found to consist of two fundamental bonding types, namely ordinary 2c/2e covalent bonds and 3c/4e hyperbonds. The latter hyperbonds successfully elucidate the formation mechanism of linear triatomic bonding motifs in *a*-GST via an LP-antibonding (hyperbonding) interaction; the triatomic bonding motif thus consists of a pair of hyperbonds. Supposing the above results to be generally applicable, we deduce from the atomic configurations of the structural units in Fig. 3 that octet units are composed only of ordinary covalent bonds, whereas every hypervalent unit consists of a mixture of ordinary covalent bonds and triatomic hyperbond pairs, as illustrated in Figs. 1 and 2 (except for the rare 6-coordinated unit which only has three hyperbond pairs). The classification into two chemical-bonding types in such amorphous solids is clearly verified by the following material-independent observations (Fig. 3). Firstly, the hyperbonds tend to exhibit, on average, longer bond lengths (${\bar{r}}_{{{{{{\rm{hyp}}}}}}}$) than covalent bonds (${\bar{r}}_{{{{{{\rm{cov}}}}}}}$), by ~5% (as inferred from $\Delta {\bar{r}}_{{hyp}-{cov}}$), while also being more polarised (as evidenced by a larger mean polarity index, *χ*) and more delocalised (as measured by the mean spread of MLWFs, ${\bar{\Omega }}^{1/2}$, and the positive values of $\Delta {\bar{\Omega }}_{{hyp}-{cov}}^{1/2}$). Secondly, the hyperbonds are generally weaker in bond strength, presumably with a bond order much smaller than for ordinary covalent bonds^37^, as indicated from the longer bond lengths and smaller values of the electron localisation function (ELF) at bond critical points (see Table 1). Thirdly, no chemical distinction between the covalent bonds in hypervalent units and in octet-conforming units is apparent, as inferred from near-zero values of the quantity $\Delta {\bar{r}}_{{cov}-{cov}* }=({\bar{r}}_{{cov}}/{\bar{r}}_{{cov}* }-1)\times 100$, where ${\bar{r}}_{{cov}* }$ denotes the average bond length of either octet (2,2)-type units for *a*-Te, or octet (3,1)-type units for the other models. The above observations may then rationalise our universal classification of not only chemical interactions into two distinguishable types (i.e. covalent bonds and hyperbonds), but also structural units into octet and hypervalent units for a diversity of amorphous chalcogenide solids. It should be noted that there also exist some tiny populations of unit types, not listed in Fig. 3. Those units could be considered as defect configurations, and were not considered here.

**Table 1:** Comparison of structural units and their properties in models of amorphous chalcogens and ternary chalcogenides.

Model	Structural unit (CN, # LP)	Octet/hyperv.	Z* (e)	Bonding type	*r* (Å)	ELF_b_	*χ* (polarity index)	MLWF_bond_ spread ${\Omega }^{1/2}$ (Å)
*a*-S	S(2,2)	Octet	−0.01 (0.14)	Covalent	2.07 (0.07)	0.8 (0.03)	0.50 (0.00)	1.08 (0.04)
*a*-Se	Se(2,2)	Octet	0.00 (0.38)	Covalent	2.39 (0.04)	0.73 (0.01)	0.50 (0.01)	1.31 (0.03)
*a*-Te	Te(2,2)	Octet	−0.15 (1.57)	Covalent	2.89 (0.07)	0.67 (0.05)	0.50 (0.06)	1.72 (0.10)
	Te(3,1)	Octet	−0.62 (1.54)	Cov. + Hyp.	2.97 (0.10)	0.63 (0.06)	0.50 (0.10)	1.77 (0.10)
	Te(3,2)	Hyperv.	1.44 (1.02)	Covalent	2.90 (0.07)	0.68 (0.04)	0.49 (0.06)	1.72 (0.09)
				Hyperbond	3.05 (0.06)	0.57 (0.04)	0.64 (0.03)	1.84 (0.07)
*a*-GSS	Ge(3,1)	Octet	2.14 (0.34)	Covalent	2.47 (0.07)	0.68 (0.05)	0.69 (0.03)	1.30 (0.03)
	Ge(4,0)	Octet	2.25 (0.39)	Covalent	2.31 (0.05)	0.81 (0.03)	0.62 (0.03)	1.26 (0.03)
	Sb(3,1)	Octet	2.82 (0.70)	Covalent	2.52 (0.07)	0.71 (0.05)	0.65 (0.03)	1.31 (0.03)
	Sb(4,1)	Hyperv.	3.13 (0.78)	Covalent	2.53 (0.07)	0.72 (0.05)	0.65 (0.03)	1.31 (0.02)
				Hyperbond	2.77 (0.05)	0.54 (0.03)	0.74 (0.02)	1.37 (0.02)
*a*-GSSe	Ge(3,1)	Octet	2.13 (0.42)	Covalent	2.61 (0.07)	0.69 (0.05)	0.68 (0.03)	1.44 (0.04)
	Ge(4,0)	Octet	2.22 (0.39)	Covalent	2.46 (0.05)	0.79 (0.03)	0.60 (0.03)	1.39 (0.03)
	Sb(3,1)	Octet	2.74 (0.75)	Covalent	2.67 (0.07)	0.72 (0.04)	0.63 (0.03)	1.45 (0.04)
	Sb(4,1)	Hyperv.	3.66 (0.55)	Covalent	2.68 (0.07)	0.73 (0.04)	0.63 (0.03)	1.45 (0.05)
				Hyperbond	2.90 (0.06)	0.56 (0.04)	0.73 (0.02)	1.53 (0.03)
*a*-GST	Ge(3,1)	Octet	2.76 (0.85)	Covalent	2.82 (0.07)	0.69 (0.05)	0.65 (0.04)	1.74 (0.08)
	Ge(4,0)	Octet	2.07 (0.80)	Covalent	2.73 (0.07)	0.75 (0.05)	0.59 (0.04)	1.68 (0.07)
	Ge(4,1)	Hyperv.	3.60 (0.92)	Covalent	2.86 (0.08)	0.67 (0.07)	0.67 (0.04)	1.77 (0.10)
				Hyperbond	2.98 (0.06)	0.56 (0.05)	0.73 (0.03)	1.86 (0.11)
	Sb(3,1)	Octet	3.10 (1.63)	Covalent	2.92 (0.06)	0.71 (0.04)	0.60 (0.03)	1.73 (0.07)
	Sb(4,1)	Hyperv.	4.56 (1.45)	Covalent	2.92 (0.06)	0.71 (0.04)	0.60 (0.03)	1.73 (0.08)
				Hyperbond	3.10 (0.06)	0.57 (0.04)	0.70 (0.03)	1.88 (0.09)
	Sb(5,1)	Hyperv.	5.24 (1.49)	Covalent	2.94 (0.05)	0.71 (0.04)	0.60 (0.03)	1.79 (0.07)
				Hyperbond	3.09 (0.07)	0.58 (0.05)	0.70 (0.03)	1.89 (0.09)

Structural units conforming to the octet rule (‘octet’) have four pairs of electrons in total, whilst more than four pairs are associated with hypervalent units (‘hyperv.’). The structural units for cations for ternary chalcogenides only are shown. The standard deviation for each observable is shown in parentheses. Z* indicates the Born effective charge, *r* the bond length and ELF_b_ is the ELF value at a bond-critical point. The spread of MLWF_bond_ corresponds to ${\Omega }^{1/2}$ (see text) for either covalent bonds or hyperbonds. $\bar{\chi }$ denotes a polarity index, as defined in Lee and Elliott^4^, quantifying the ionicity of a bond; for instance, $\bar{\chi }$ is supposed to be 0.5 for homopolar bonds, and a positive deviation from this value denotes the degree of charge transfer of bonding electrons to a ligand. For a proper comparison, we present chemical-bonding properties of chemically-correct bonds with only chalcogen ligands for each structural unit. The dist*r*ibutions of *r*, ELF_b_, *χ* and the spread of MLWF_bond_ values for selected hypervalent units are shown in Supplementary Figs. 6–9.

The material-dependent population of hypervalent units naturally follows the general rule governing hyperbond formation. According to the hyperbonding model^28^, the probability of hyperbond formation scales inversely with the band gap of the amorphous solid. This is indeed what is observed in this study: a larger population of hypervalent units is observed in narrower band gap materials, e.g. the tellurides *a*-Te or *a*-GST, than in larger band-gap sulfide or selenide materials (see Figs. 1 and 2).

For completeness, some brief notes on the chemical-bonding properties of the octet, tetrahedral Ge(4,0) units can be made. This type of unit is distinct from the others in the sense that no LP is present at the central Ge atom, and it also tends to make a ‘wrong’ bond with another cation, as already noted before. Also, because of the more *s*-*p* hybridised AOs of the central Ge atom, Ge-Te covalent bonds in Ge(4,0) units exhibit, in comparison with those in other Ge units, the shortest bond length, the strongest bond strength, and are the least polarised, as indicated in Table 1.

### Relation between hypervalent units and abnormally large Born effective charges

Owing to the characteristic bonding types and geometries for each type of structural unit, microscopic material properties are often strongly dependent on the types of units present. One such property that we investigated is the Born effective charge (BEC). As shown in Fig. 3, the central atoms of octet-conforming units display mean values of BEC close to their nominal ionic charges. By comparison, values of BECs much larger than the nominal charges of the atoms are generally found for hypervalent units. An example best reflecting this behaviour would be the structural units in *a*-Te. The mean BEC value for the octet Te(2,2) units amounts to −0.15, which is close to its zero nominal ionic charge, whereas a marked deviation in BEC is observed for the hypervalent unit Te(3,2), with a BEC value of +1.44. The other octet-conforming unit, Te(3,1), that is preferentially bonded to the Te(3,2) unit as a ligand along the hyperbonding direction, exhibits a negative mean BEC value of −0.62. The Te-Te-Te triatomic hyperbond pair is thus composed of three Te atoms that show, on average, significant anomalous BEC contributions with alternating signs, viz. a positive contribution (+1.44) for the central Te atom and a negative one (−0.62) for the Te ligands, while overall satisfying the sum rule for charge neutrality^38^. This array of atoms with significantly anomalous BECs is clearly indicative of the large polarizability of hyperbonds. According to Harrison^39^, an alternative physical interpretation of the large BEC value associated with the hypervalent units could be a significant change in orbital hybridisation that leads to considerable charge transfer between the atoms constituting the hyperbond pair, with respect to a small displacement of the central Te atom. Both of these interpretations are, in principle, consistent with the viewpoint of the multi-centre hyperbonding concept: the displacement of a central atom along the axis of a triatomic hyperbond pair corresponds to a reverse of the hyperbonding process via the bond-breaking transition from a triatomic hyperbonding pair (Te-Te-Te) to a Te LP (Te:) plus an ordinary covalent bond (Te-Te), which should be accompanied by considerable changes in orbital hybridisation and charge transfer.

**Fig. 4:**
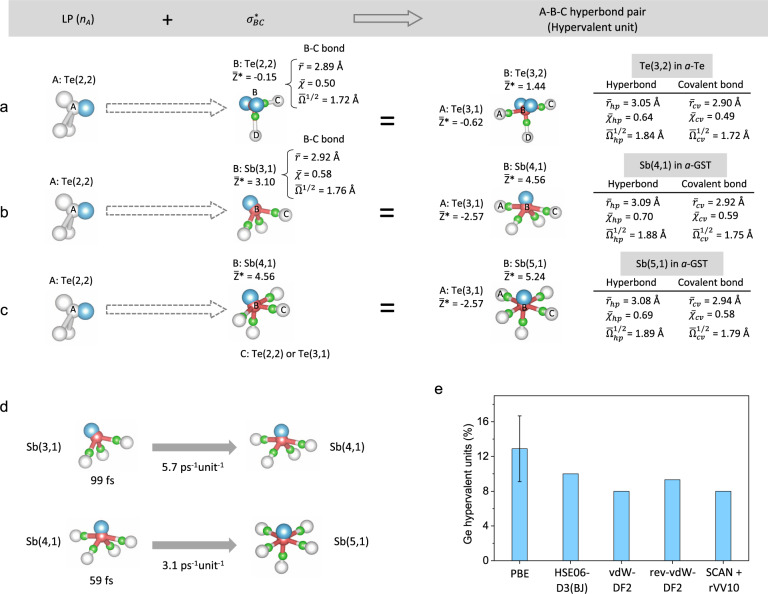
Proposed formation processes of representative hypervalent units. The hyperbonding interactions of an LP of the central Te (A) atom of a (2,2)-type unit with an antibonding orbital of a nearby bond (B-C) of: **a** Te(2,2) in *a*-Te; **b** Sb(3,1) in *a*-GST; and **c** Sb(4,1) in *a*-GST. For each hypervalent unit, its corresponding mean Born effective charge, as well as the properties of their covalent bonds and hyperbonds, are compared in **a**–**c**. The A-B-C triatomic bonding configuration consists of a hyperbond pair (A-B and B-C). A central atom (B), together with corresponding MLWF centres for LPs (cyan) and bonds (green), constitutes each structural unit. **d** The lifetime of Sb(3,1) and Sb(4,1) units, and their transition rate (per ps per unit) to the associated hypervalent unit in *a*-GST at 700 K, calculated from the trajectories of structural units in AIMD simulations. **e** Variation of the population of Ge hypervalent units in *a*-GeTe with the inclusion of various dispersion-interaction corrections^40–43^. The error bar for the PBE functional represents the standard deviation among independently-generated *a*-GeTe models.

Taken together, it is the high polarizability of hyperbonds, or associated charge transfer with respect to a displacement of a central atom, that leads to large positive BEC values of hypervalent units. The same reasoning can apply to cation-centred hypervalent units in binary or ternary chalcogenide alloys, in which a central cation (i.e., Ge or Sb) and electronegative Te ligand anions are now involved in bonding. The cation-centred hypervalent units exhibit, in common, large positive BECs compared to their nominal ionic charges due to the presence of hyperbonds, as in the case of the hypervalent units in *a*-Te. Sb-centred hypervalent units also show, on average, a larger BEC than Ge-centred counterparts. It should be noted, however, that, since we define the BEC for each atom as one-third of the trace of its BEC tensor, the larger mean BEC value for (5,1) than for (4,1) hypervalent units may arise simply due to averaging over two hyperbonding directions rather than one for the (4,1) units. In any case, because of the different BEC values for different structural units, it is expected that optical properties of amorphous materials depend on the ratio of the number of hypervalent to octet units, although their spatial alignments are also of direct relevance to this property. A relatively extreme example of this kind is the crystalline state of the phase-change material, GST, with a large dielectric constant^5^, whose structural models indicate that 80% (respectively 98%) of Ge (or Sb) atoms adopt a certain type of hypervalent unit with slightly distorted cubic crystalline symmetries (see Supplementary Fig. 3). The origin of the large dielectric constant of the crystalline phase-change materials, and the large optical contrast with their amorphous counterparts, thus can be accounted for in the theoretical framework of hypervalency supplemented by a structural-unit description of both amorphous and crystalline materials. The phase-transition or kinetic properties of their supercooled or liquid phases are also linked to the dynamical behaviour of octet or hypervalent units (as inferred in Fig. 4) with varying transition rates^4^, providing rich future research directions related to a diversity of material properties.

### Formation mechanism of hypervalent units

The astonishing contrast in BEC values and chemical properties between octet and non-octet units described so far becomes more obvious if one considers the following formation process proposed for hypervalent units. Figure 4a describes two simultaneous transition processes of Te_A_(2,2) → Te_A_(3,1) (process I) and Te_B_(2,2) → Te_B_(3,2) (process II) in *a*-Te, where the subscript letter denotes the identity of Te atoms shown in Fig. 4a. As indicated in the figure, process II for the octet-to-hypervalent transition is accompanied by process I for the octet-to-octet transition, overall producing a single hypervalent Te(3,2) and an octet Te(3,1) unit from two starting octet Te(2,2) units. The chemical-bonding properties and BECs are accordingly altered. More precisely, as a Te_B_-Te_C_ homopolar bond with $\bar{\chi }$ = 0.50 transforms to a Te_A_-Te_B_-Te_C_ triatomic hyperbond pair via hyperbonding (an interaction of an LP (cyan in Fig. 4a–c) of Te_A_ with an antibonding orbital ${\sigma }_{{BC}}^{* }$ of Te_B_-Te_C_), the Te_B_-Te_C_ bond abruptly changes to become a longer (weaker) polar bond with $\bar{\chi }$ = 0.64. At the same time, the near-zero BEC value of the central Te_B_ atom (respectively, ligand Te_A_) becomes large and positive (or negative), respectively, an indication of enhanced polarizability along the axis of the hyperbonded pair. Interestingly, despite significant changes in bonding along the hyperbonding direction, the adjacent covalent bond (Te_B_-Te_D_) is largely unaffected by this transformation. The formation processes of the other two hypervalent units of (4,1) and (5,1) types can be similarly described by employing hypervalent Sb units, as observed in *a*-GST. The hypervalent Sb(4,1) unit can form as a result of two simultaneous transitions (Fig. 4b): Te(2,2) → Te(3,1) and Sb(3,1) → Sb(4,1). The coupled transitions for hypervalent Sb(5,1) units (Fig. 4c) correspond to Te(2,2) → Te(3,1) and Sb(4,1) → Sb(5,1) transformations. One can notice from these two processes (Fig. 4b, c) that an Sb(5,1) unit can be constructed from an Sb(3,1) unit by two successive hyperbonding interactions along two perpendicular hyperbonding directions. The subsequent changes in average bond length ($\bar{r}$), polarity index ($\bar{\chi }$), MLWF delocalisation ($\bar{\Omega }$^1/2^), and BEC value are largely consistent with the trend predicted by the hyperbonding model, as indicated in Fig. 4a. The formation mechanisms proposed for the hypervalent units in Fig. 4a–c are directly verified from the AIMD simulations, wherein their formation mechanisms are fully explored in terms of the evolution of structural units at elevated temperatures. At 700 K, the average lifetimes of Sb(3,1) and Sb(4,1) units in *a*-GST were found to be 99 and 59 fs, respectively, and they transform, as suggested in Fig. 4b, c, to the hypervalent units of Sb(4,1) and Sb(5,1) with a transition frequency of 5.7 and 3.1 ps^−1^unit^−1^, respectively (Fig. 4d). The formation of hypervalent Te(3,2) units in *a*-Te (Fig. 4a) is similarly directly observed from AIMD simulations at elevated temperatures.

It should be noted that, in every formation process, a Te(2,2) unit plays the role of donating an LP for hyperbonding, while transforming to a Te(3,1) unit. For this reason, the transition from Te(2,2) to Te(3,1) units (process I) is considered to be important for hypervalent-unit formation in amorphous tellurides, as well as in *a*-Te itself. The observed qualitative proportional relationship between the populations of Te(3,1) and hypervalent units (see Figs. 1 and 2) is precisely in accord with this interpretation. A similar conclusion is also reached for sulphur or selenium (3,1)-type units in *a*-GSS or *a*-GSSe, respectively, emphasising the general role of chalcogen LPs in hyperbonding and hypervalent-unit formation.

Unlike for the other models, the population of Te(3,1) units in *a*-GeTe is much higher than in any of the other chalcogenides studied (Fig. 2a). This may be associated with not only its anion-deficient composition, but also the fact that, as inferred from its electronic configuration (4*s*^2^*p*^2^), a Ge atom needs to form a dative bond with a nearby Te atom in order to retain even its most stable, abundant type of unit, *viz*. Ge(3,1). The formation of dative bonds hence leads to an increase of the Te(3,1) population at the expense of that of Te(2,2) units. In contrast, Sb atoms (5*s*^2^*p*^3^) do not require such a dative bond for the formation of Sb(3,1) units, which leads to a much lower Te(3,1) population observed in Sb-containing chalcogenides (Fig. 2).

Now, it is instructive to consider the effect of van der Waals (vdW) interactions on hypervalency. To this end, we focused on the impact of including vdW interactions on the population of hypervalent units in models of *a*-GeTe, as a representative example, by considering various computational methods developed to account for vdW interactions in the framework of Kohn-Sham DFT simulations: dispersion corrections via the DFT-D3 method^40^ or the inclusion of a non-local correlation functional into the (semi-)local exchange and correlation functionals^41–43^. Figure 4e shows computed populations of hypervalent Ge units (*viz*. Ge(4,1) and Ge(5,1)) in models of *a-*GeTe, simulated with vdW interactions being included. Although only a relatively limited number of models were considered, it appears that the percentage of hypervalent Ge units in the models tends to decline very slightly with the inclusion of vdW interactions, but still remains close to the lower limit of the error bar computed for only the semi-local PBE functional.

### Revised hyperbonding model

**Fig. 5:**
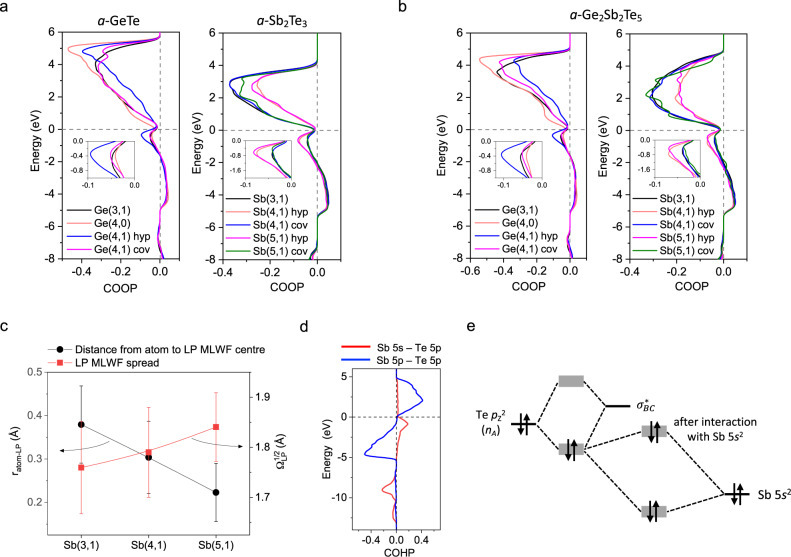
Revised hyperbonding model involving cation *s*^2^ LPs. Crystal-orbital overlap populations (COOPs) for the chemical bonds of structural units in: **a** binary *a*-GeTe and *a*-Sb_2_Te_3_; and **b** ternary *a*-GST. The Fermi level is indicated by the horizontal dashed line. The magnified COOP data near the Fermi level are shown in the inset. The abbreviations ‘hyp’ and ‘cov’ denote hyperbonds and covalent bonds, respectively, for each type of structural unit. A clear distinction between the hyperbonds and covalent bonds is made from their COOP profiles. **c** Distance distributions for a central atom (Sb) and MLWF_LP_ centres for different units in *a*-GST. The spreads of MLWF_LP_ (${\Omega }_{{LP}}^{1/2}$) values are also shown. **d** Crystal-orbital Hamilton populations (COHPs) for the hyperbonds of Sb(4,1) units in *a*-GST. Orbital-resolved COHP*s* for Sb 5 *s* – Te 5*p* (*s*-*p*) and for Sb 5*p* – Te 5*p* (*p*-*p*) are depicted. Note the antibonding character (positive COHP value*s*) for Sb 5 *s* – Te 5*p* states at the top of the valence band. **e** A schematic interaction-energy diagram for an LP (*n*_A_) stabilisation (hyperbonding) interaction with a nearby antibonding orbital (${\sigma }_{{BC}}^{* }$) and with an Sb 5*s*^2^ (LP) orbital.

A very curious observation about the electronic structure of amorphous and crystalline chalcogenide PCMs is that crystal-orbital overlap population (COOP) or crystal-orbital Hamilton population (COHP) analyses^44,45^ reveal a universal, pronounced antibonding character (i.e. negative COOP or positive COHP values) of electron states lying at the top of the valence band (VB), below the Fermi level, where it would normally be expected that the states would have bonding character (i.e. positive COOP or negative COHP values) or non-bonding (*p-*like LP) character (near-zero COOP or COHP values)^4,46,47^. As shown in Fig. 5a, b, the model systems considered here also reveal an antibonding character near the top of the VB (see Supplementary Fig. 4). In particular, more pronounced antibonding peaks are observed for the hyperbonds of hypervalent units compared to those of any covalent bond. The origin of such antibonding states in the VB, normally to be expected only in the conduction band, has not previously been clear. Indeed, an orbital-resolved COHP analysis for *a*-GST (Fig. 5d) shows that most of the antibonding states at the top of the VB originate from an (*s*-*p*) interaction between cation *s* and anion *p* atomic orbitals (AOs), implying a non-negligible involvement of cation LPs in the overall interatomic interactions. The changes in MLWFs provide a simple idea concerning these observations (Fig. 5c). As an Sb(3,1) unit, for instance, successively transforms to an Sb(4,1) and then to an Sb(5,1) unit, the (*s*^2^) LP of the central Sb atom exhibits a clear sign of expansion (or delocalisation), along with a shortening of the distance between the Sb atom and the LP (*r*_atom-LP_), as shown in Fig. 5c. The latter observation of decreasing *r*_atom-LP_ values means a successively diminishing *s-p* hybridisation of the Sb AOs involved in bonding and LPs. These changes likely induce a stronger orbital overlap between the LP and hyperbonds, and thus stronger orbital interactions between them. The interaction pattern of the cation LPs with hyperbonds generally differs from that for ordinary covalent bonds. This difference is inferred from the observation that the former interaction between the cation *s* and anion *p* states, comprising hyperbonds, leads to a sharp *s-p* antibonding peak right below the top of the VB, whereas the latter interaction gives rise to a much wider, flatter one, distributed over the valence and unoccupied conduction bands (see Supplementary Fig. 5). This trend is universally observed, independent of material type.

To incorporate this extra interaction into the original hyperbonding model^28^, we here propose a revised model, as schematically depicted in Fig. 4e. According to this revised model, additional bonding and antibonding states appear as a result of the interaction of hyperbonding states with deep-lying cation *s*^2^ LP orbitals. The resulting, filled, antibonding states occur just below the top of the valence band, and contain a considerable amount of cation *s* and anion *p* character, thereby explaining the anomalous COHP results (see Fig. 4e). Because of the narrow uppermost anion LP band (n_a_) involved in this interaction, a narrow *s*-*p* antibonding band would result at the top of the VB. A similar interaction diagram for covalent bonds again yields a large cation *s* character at the top of the VB (Supplementary Fig. 5). In this picture, however, the interacting anion states are now from the wider anion *p* band below the LP band; the resultant *s*-*p* antibonding band arising due to the interaction between a *p-p* bonding state, σ_*pp*_ and a cation *s*^2^ LP is much wider than that for the hyperbonds. The influence of the extra interaction with an anion *s* orbital may then be different, since the associated *s*-*p* antibonding states for the ordinary covalent bonds are only partially occupied with this interaction (Supplementary Fig. 5), while those for the hyperbonds are nearly fully occupied. It is noted that the cation *p* – anion *p* interaction for the hyperbonds also often leads to antibonding states near the top of the valence band (Fig. 4e), but this is in general much less significant than the *s*-*p* interaction. The involvement of cation *s-*like LP AOs in interatomic interactions has been previously considered, in a different context, in investigating cation *s*^2^ – LP stereochemical effects, for crystalline chalcogenides, by Waghmare et al.^46^ (who also found an antibonding character from a COHP analysis for cation *s* – anion *p* states at the top of the VB in crystalline GeS, GeTe and PbTe) and for crystalline oxides (e.g. PbO) by Walsh et al.^48^ A remaining question is then whether there is any possible impact on microscopic material properties, caused by the extra interaction with a cation *s*^2^ LP, as considered in the revised hyperbonding model. We first notice that hyperbonded units in amorphous chalcogenides always appear together with the presence of a cation LP at their centre (see the hypervalent units in Fig. 3). Treating them as a single entity means that the inferred large polarizability of hyperbonds, and associated large BEC values, are the characteristics of cation LP-hyperbond complexes, not solely of hyperbonds themselves. The exact role of cation *s*^2^ LPs in this particular case, as well as in other related properties, such as phonon anharmonicity^49^, is still unclear and is an open question^50^ worthy of further study.

## Discussion

It is interesting to compare our model with the recently proposed metavalent-bonding model^51,52^, where chemical bonds in crystalline materials are categorised in terms of the amount of shared and transferred electron charges. According to the 2D ‘map’ constructed with these chemical parameters, an average bond order (centred around ~0.5) smaller than that of ideal covalent bonds (with a bond order of 1.0), and an average charge transfer of the order of ~0.5 e (while being spread over the range 0.3–1.0 e), are asserted to represent the characteristic bonding properties of metavalently bonded materials^51^. For a comparison independent of either of the models, we may denote this area in the map as a partially delocalised (PD) bonding region that links localised (L) covalent bonding and fully delocalised (FD) metallic bonding.

Interestingly, this characteristic range of values of the chemical parameters is similarly observed from a natural-bond-orbital (NBO) study of hypervalent molecules^29,53^, wherein the segment of linear triatomic bonds revealed similar bond orders and charge transfers corresponding to the partially delocalised (PD)-bonding area in the map. On the other hand, the other bonding segment in the molecules showed values close to that of an ideal covalent bond; in other words, both localised, and partially delocalised, bonding types coexist in the same hypervalent molecule, and the corresponding two separate regions in the map need to be simultaneously considered if such molecular systems are to be thoroughly described using such maps. Similarly, for solids with the two bonding types (other than van der Waals interactions) in their structures, anisotropic, mixed bonding configurations are widely observed, as is the case in the current study of amorphous chalcogenides, which can be regarded as a condensed solid form of molecular structural motifs. The application of this bonding concept to crystalline chalcogenides was proven to be useful in a recent comparative study of *a*- and *c*-PCMs^28^, inferring the structure-property relationship that links the linear multi-centre configurations with PD bonding and material properties (such as optical or thermal-transport properties). The illustration of PD bonding-mediated inter-unit transitions (Fig. 4) and the accompanying remarkable property changes (Figs. 3 and 4) highlight the broad conceptual applicability of varying degrees of electron delocalisation beyond two-centre bonding to the study of the static and dynamic behaviour of solid material systems.

Now, it becomes clear that, for our purpose, the chemical-bonding theory required should be capable of (i) covering the structural range from small molecules to extended amorphous/crystalline solids; (ii) dealing with the coexistence of variable degrees of delocalisation of bonding/non-bonding electrons; (iii) elucidating the mechanism of PD-L bonding transitions; and (iv) providing a theoretical account of the microscopic origin of structure-property relationships. We argue that all these interesting aspects of chemical-bonding interactions can be comprehensively elucidated using the theory of hypervalency that the current study provides. The concept of hypervalency that blends all the ingredients listed above into a single unifying theoretical framework is thus rationalised by its simple atomic-orbital-based description, wide applicability and potential usefulness in developing materials based on the structure-property relationships that the theory provides, which are lacking in the other bonding models proposed so far.

In conclusion, hypervalency constitutes an indispensable and important element in understanding chemical-bonding networks of amorphous materials, in particular where a strong propensity for multi-centre hyperbonding interactions exists among the constituent elements, such as in amorphous chalcogenides, especially tellurides. A manifestation of hypervalency is the presence of non-electron-octet-conforming hypervalent units, with the number of associated LPs and bonding electron pairs exceeding four. The general coordination geometry of each hypervalent unit takes a particular shape, merely depending on their numbers of associated LPs and bonding electron pairs, and follows the VSEPR theory. The observation of abnormally large Born effective charges of hypervalent units is closely related to the large polarizability of (near-) linear triatomic hyperbond pairs, the basic characteristic of hypervalent units, differentiating them from octet units. This study reinforces the structure-property relationship proposed previously^28^ between a linear triatomic hyperbonding geometry and large property contrasts between amorphous and crystalline telluride chalcogenides, while broadening the scope of this relationship to amorphous chalcogen and other binary/ternary chalcogenides as well, thereby providing a unified theoretical framework, useful for understanding the structure and properties of amorphous network materials in general.

## Methods

### Ab initio molecular-dynamics (AIMD) simulations

For the generation and analysis of amorphous models, ab initio molecular-dynamics (AIMD) simulations based on density-functional theory (DFT) were employed, as implemented in the Vienna Ab initio Simulation Package (VASP) code^54^. For all amorphous models considered here, we consistently used the Perdew-Burke-Ernzerhof (PBE) exchange-correlation functional^55^ with the projector augmented-wave (PAW) method^56^ for a proper comparison between models. To investigate the effect of dispersion (van der Waals) interactions on hypervalent units, four exchange-correlation functionals, HSE06-D3(BJ)^40^, vdW-DF2^41^, rev-vdW-DF2^42^ and SCAN + rVV10^43^, were additionally employed during the generation and analysis of *a*-GeTe models. The plane-wave energy cutoff used was 300 or 400 eV. All the outer *s* and *p* electrons of constituent atoms were treated as valence electrons. The Brillouin zone was sampled at the Γ point only. The temperature was controlled by a Nosé-Hoover thermostat algorithm, and the MD time step was 2–4 fs. The amorphous models were simulated in cubic supercells with periodic boundary conditions. The volumes of models were kept constant during the AIMD simulations. The conjugate-gradient method was employed for structural relaxation of quenched amorphous models. Structural relaxation was performed until the force on any atom was less than 0.01 eV/Å. Density-functional perturbation theory was used to calculate the Born effective charges (BECs) of constituent atoms, as implemented in VASP^54^.

### Amorphous models

Amorphous models were generated following the conventional melt-quench method. For the amorphous chalcogen models, 300 atoms were randomly mixed at 2000 K in a cubic cell for a few ps. Then, the models were equilibrated at temperatures higher than their melting temperatures. For *a*-S, the model was kept at 473 K for 35 ps, and then quenched to 0 K with a quench rate of −15 K/ps. This quench rate and equilibration time of tens of ps were used in common for the other models. The equilibration temperature for the other chalcogen models was 650 K or 900 K for *a*-Se or *a*-Te, respectively. All the quenched amorphous chalcogen models were then relaxed, and their structures and chemical-bonding properties were characterised. Models of the binary amorphous tellurides, *a*-GeTe and *a*-Sb_2_Te_3_, were similarly generated. The equilibration temperatures for their liquid phases were 1073 K or 1000 K for *a*-GeTe (300 atoms) or *a*-Sb_2_Te_3_ (320 atoms), respectively. The ternary amorphous chalcogenides chosen for this study were *a*-Ge_2_Sb_2_S_5_ (*a*-GSS), *a*-Ge_2_Sb_2_Se_5_ (*a*-GSSe) and *a*-Ge_2_Sb_2_Te_5_ (*a*-GST). For these ternary models (315 atoms), the equilibration temperature was 900 K. The densities used for the above ternary models were 3.43, 4.41 and 5.88 g/cm^3^, respectively. We generated three independently-quenched amorphous models for each binary and ternary composition for better statistics.

### Structural units and chemical-bonding properties

To define structural units, both MLWF and ELF analyses were used simultaneously. MLWFs were constructed from a unitary transformation of Kohn-Sham orbitals, and then maximally localised to generate MLWF centres using the Wannier90 code^57^. The MLWF centres were then classified as representing either a bond or a lone pair (LP), depending on whether an MLWF centre is associated with a chemical interaction between a pair of atoms strong enough to be defined as a bond. Instead of the quadratic spread (Ω) of MLWF produced by the Wannier90 code, we used Ω^1/2^ values to represent the delocalisation of MLWFs. The ELF value at a bond-critical point (ELF_b_), determining the formation of an ordinary covalent bond, was chosen to be 0.5^4^. The ELF_b_ values were calculated within the framework of Quantum Theory of Atoms in Molecules (QTAIM)^58^. A hyperbond pair with a (near-) linear triatomic bonding geometry, consisting of two equivalent hyperbonds, was defined when ELF_b_ for both linear bonds exceeded a value of 0.5. Although we used only ELF_b_ as a chemical-bonding indicator, other indicators, such as the integrated crystal-orbital Hamilton population (ICOHP)^45^ or the energy density at bond-critical points, can be similarly employed, since there exist certain proportional relationships between them^28^. In this way, a diversity of octet-conforming and hypervalent units were determined, and then their bonding properties, as well as the Born effective charges, were characterised. The orbital-resolved COHP curves were computed using the LOBSTER code^45^. The lifetimes of hypervalent units, and the associated frequency of transitions between units, were calculated from the evolution of structural units during AIMD simulations at 700 K. Structural units, identified from ELF attractor analyses^59^, for 8000 configurations with an inter-configurational sampling interval of 8 fs (which corresponds to 64 ps of MD trajectories) were considered for the lifetime and transition-frequency calculations.

## Supplementary information

Supplementary information

## Data Availability

Information related to the structural models and analysis results are available in the Cambridge University data repository.
