# Supplementary material for: Hypervalency in amorphous chalcogenides
Source: Nat Commun. 2022 Mar 18;13:1458. doi: 10.1038/s41467-022-29054-5 (PMC8933559; doi:10.1038/s41467-022-29054-5)
Supplement: Supplementary file 1 — Supplementary information [file 41467_2022_29054_MOESM1_ESM.pdf]

Supplementary Information

Hypervalency in amorphous chalcogenides

T. H. Lee *et al.*

Supplementary Notes

Chalcogen (3,1) units: As emphasized in the main text, a lone pair (LP) of the central chalcogen atom of a (2,2) unit plays an important role in hyperbonding interactions via its stabilisation interaction with a nearby antibonding state. A byproduct of this interaction is a Te(3,1) ligand of a hypervalent unit, as illustrated in Fig. 4. Accordingly, a larger percentage of Te(3,1) ligands is expected for hypervalent units than for electron-octet units. The results in Supplementary Figure 1 exactly correspond to this expectation. In this figure, the ratio between the number of Te(3,1) and Te(2,2) ligands for the central atom (Ge or Sb) of structural units is shown. For Sb units in *a*-GST, the $CN_{Te(3,1)}/CN_{Te(2,2)}$ ratio grows from Sb(3,1) to Sb(4,1), and to Sb(5,1), as expected. The same trend of a higher $CN_{Te(3,1)}/CN_{Te(2,2)}$ ratio for hypervalent units than for octet units is also observed for Ge units, and in *a*-GSS and *a*-GSSe. For Ge units, the ratio of $CN_{Te(3,1)}/CN_{Te(2,2)}$ for Ge(3,1) units is generally much higher than that for Sb(3,1) units. As noted in the main text, this might be due to the fact that formation of a Ge(3,1) unit requires a dative bond from a nearby Te(3,1) unit at the expense of a Te(2,2) unit, which is not the case for Sb(3,1) units.

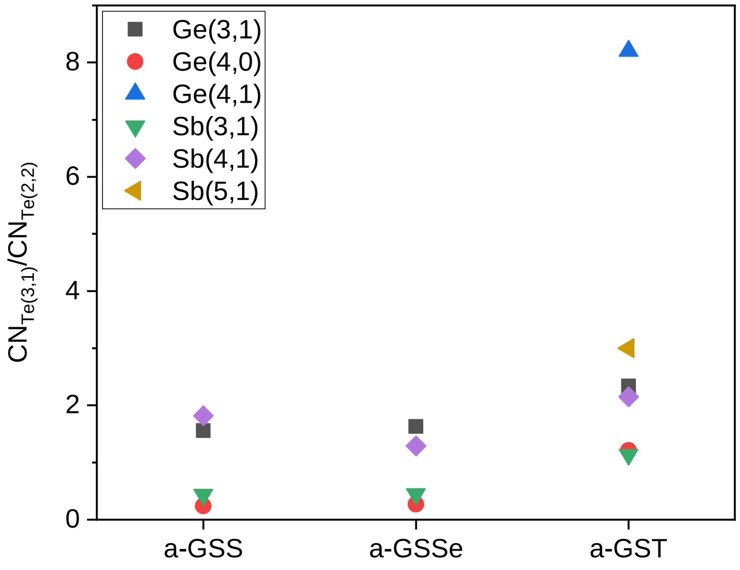

Supplementary Figure 1. The ratio of the number of Te(3,1) ligands to Te(2,2) ligands for structural units in ternary chalcogenide models. Three ternary chalcogenide models of *a*-GSS, *a*-GSSe, and *a*-GST were considered here.

Hypervalent Te(3,2) units: The 'T-shaped' molecular geometry of a (3,2)-type unit is mostly adopted by Te atoms that are coordinated mostly by Te ligands. Therefore, this type of unit is usually observed in *a*-Te (Fig. 1). However, when a locally Te-rich region happens to be present, even in multi-component amorphous tellurides, some of the Te atoms within this region tend to form (3,2)-type units. An example of a Te(3,2) unit found in *a*-GST is shown in Supplementary Figure 2. As can be seen, the central Te atom (not visible in the figure) has ligands composed only of other Te atoms (yellow).

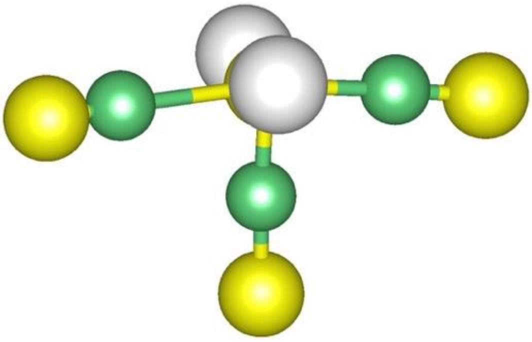

Supplementary Figure 2. A Te(3,2) unit found in *a*-GST. The positions of the MLWF centres for hyperbonds and covalent bonds (green), and for lone pairs (white), correspond to the characteristic 'T-shaped' molecular geometry of Te(3,2) units found in *a*-Te. In comparison with the covalent bond, stronger polarisations towards ligand Te atoms along the hyperbonds (i.e. a larger polarity index χ) can be noticed from the relative positions of the bond MLWF centres.

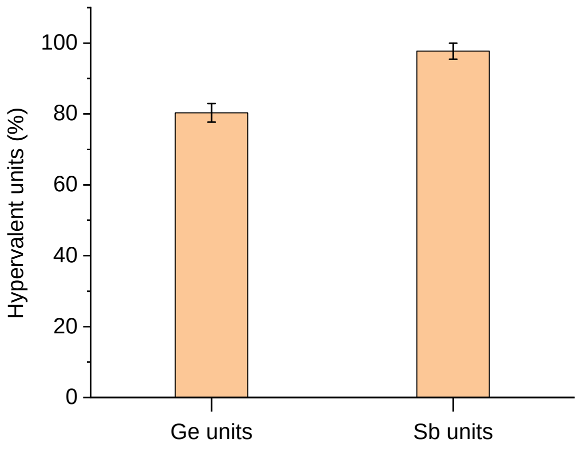

Supplementary Figure 3. The percentage of hypervalent units of Ge or Sb atoms in crystalline GST models. The error bars were taken from three metastable rocksalt-type GST models with different distributions of Ge and Sb atoms in cation sites.

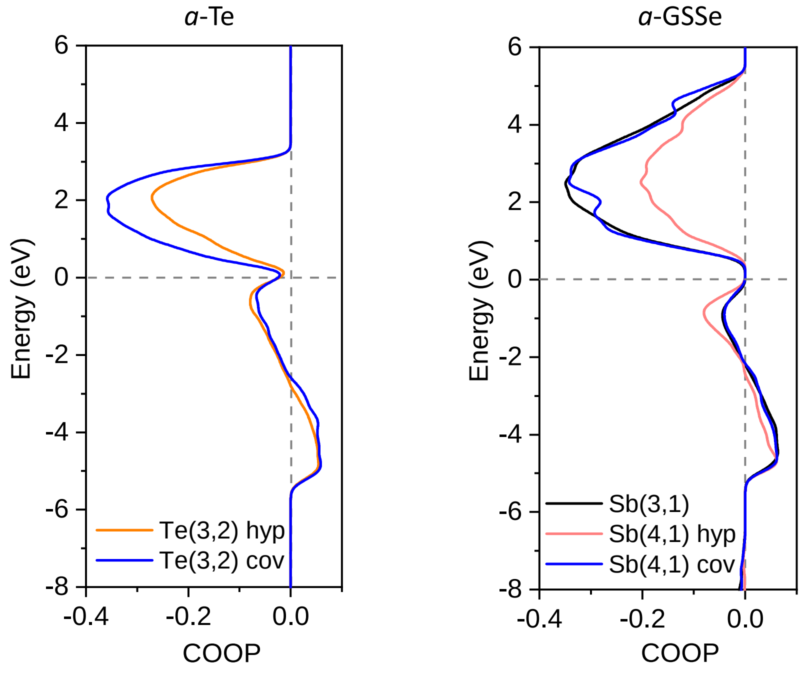

Supplementary Figure 4. Crystal-orbital overlap populations (COOPs) for the chemical bonds of structural units in *a*-Te and *a*-GSSe. The abbreviations 'hyp' and 'cov' denote a hyperbond and covalent bond, respectively, for the corresponding structural unit. For both models, the antibonding character (negative COOP values) just below the Fermi level is stronger for the hyperbonds than for covalent bonds. For a-GSSe, the similarity in COOP data between the covalent bond of octet Sb(3,1) units and those of hypervalent Sb(4,1) units is clearly shown, supporting their similar chemical-bonding nature, as claimed in the main text.

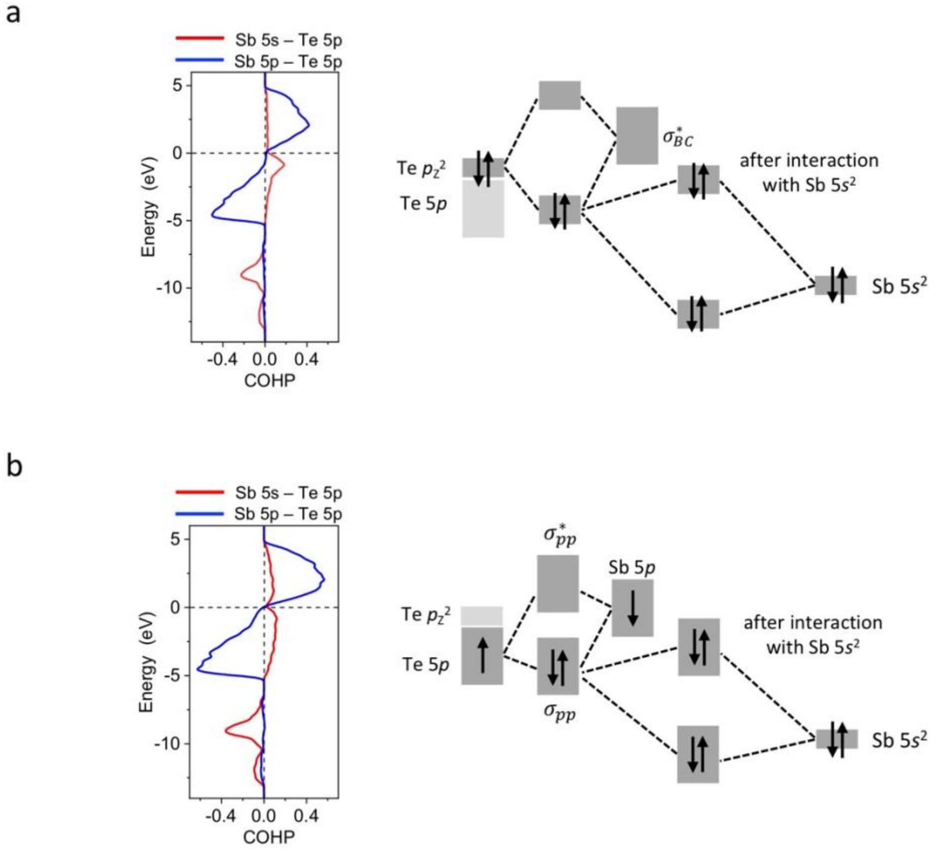

Supplementary Figure 5. Crystal-orbital Hamilton populations (COHPs) and schematic energy-band diagrams for the hyperbonds (a) and covalent bonds (b) of Sb(4,1) units in *a*-GST. Orbital-resolved COHPs for Sb 5*s* – Te 5*p* and for Sb 5*p* – Te 5*p* (*pp*) are depicted (left panels). To explain a possible origin of the difference in the cation *s* – anion *p* antibonding interactions near the top of the valence band between the covalent bonds and hyperbonds, schematic interaction diagrams of energy bands (right panels) are considered. The characteristic difference in COHP between the hyperbonds and covalent bonds (i.e. respectively narrow and wide flat antibonding *s*-*p* COHP peaks in the left panels) is commonly observed in all models considered in this study.

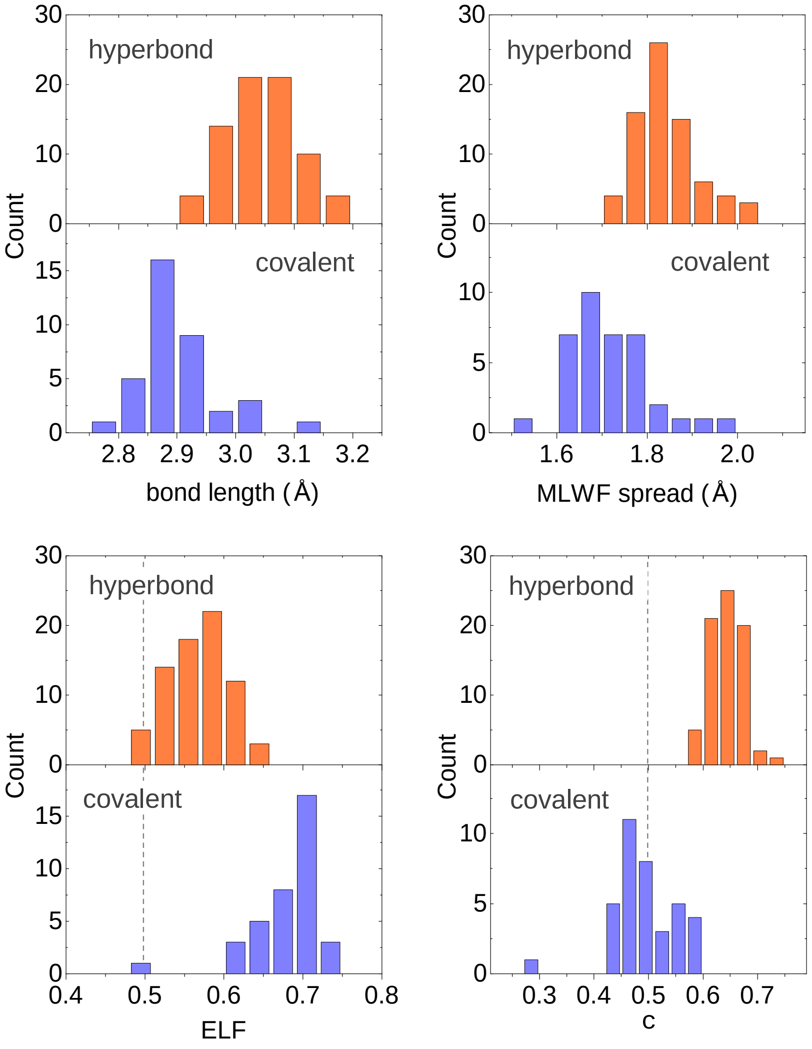

Supplementary Figure 6. Distribution of chemical-bonding properties of Te-Te bonds constituting hypervalent Te(3,2) units in *a*-Te.

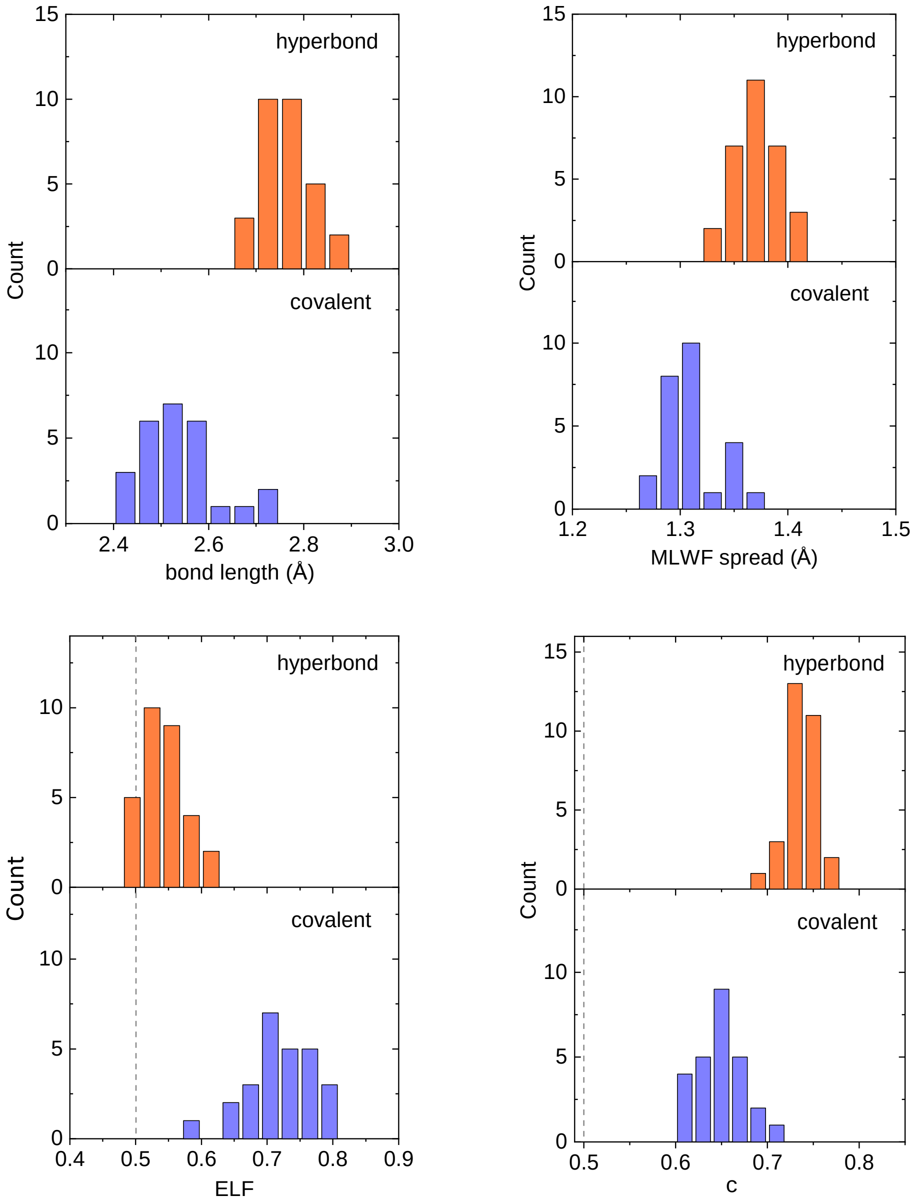

Supplementary Figure 7. Distribution of chemical-bonding properties of Sb-S bonds constituting hypervalent Sb(4,1) units in *a*-GSS.

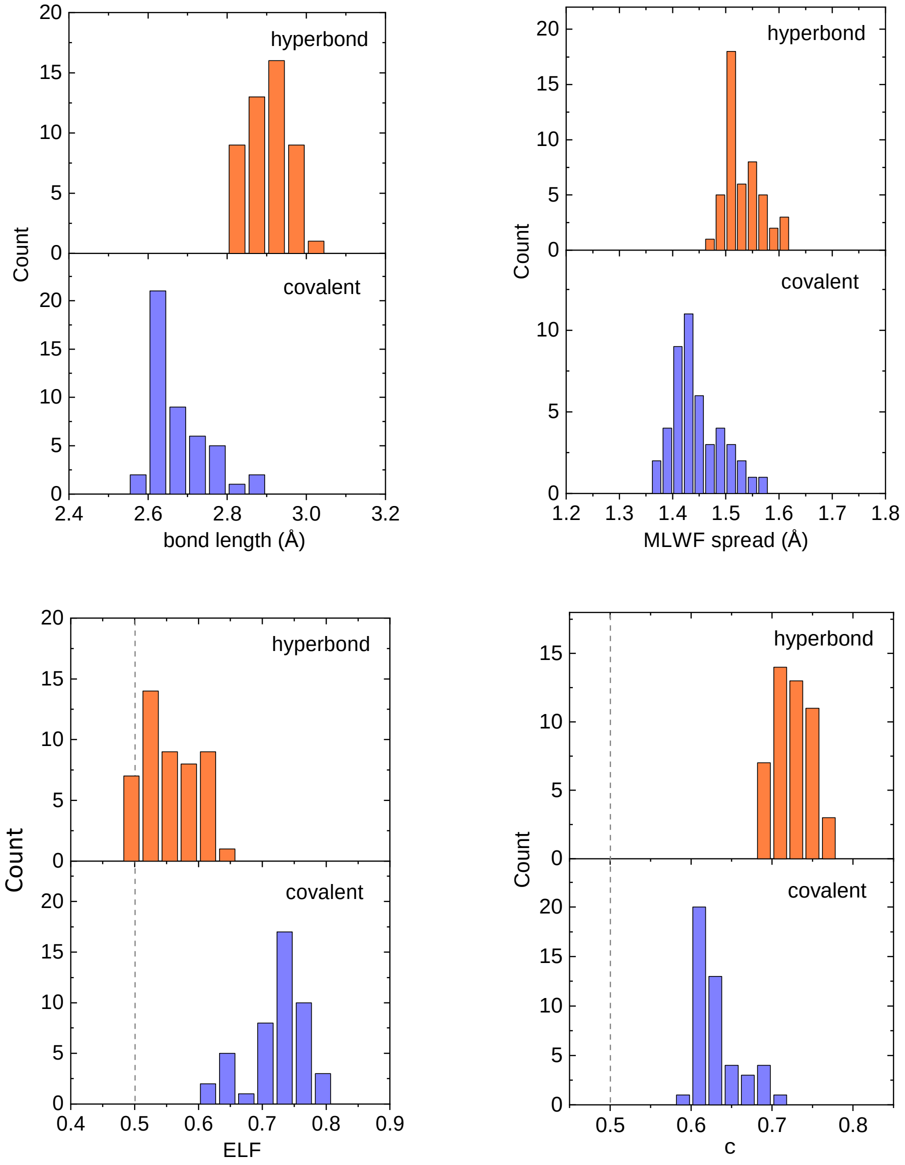

Supplementary Figure 8. Distribution of chemical-bonding properties of Sb-Se bonds constituting hypervalent Sb(4,1) units in *a*-GSSe.

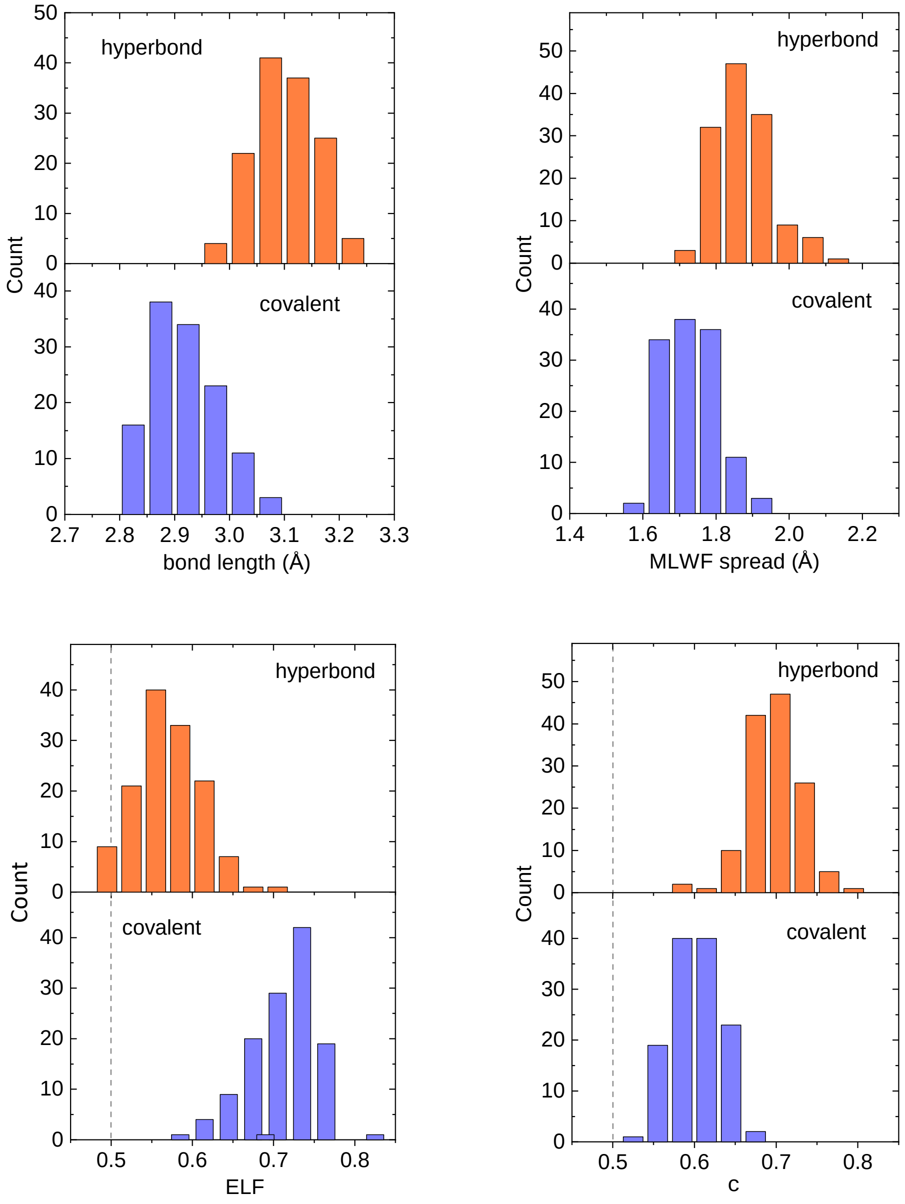

Supplementary Figure 9. Distribution of chemical-bonding properties of Sb-Te bonds constituting hypervalent Sb(4,1) units in *a*-GST.
